# Fine mapping of a QTL and identification of candidate genes associated with cold tolerance during germination in peanut (*Arachis hypogaea* L.) on chromosome B09 using whole genome re-sequencing

**DOI:** 10.3389/fpls.2023.1153293

**Published:** 2023-05-08

**Authors:** Xin Zhang, Xiaoji Zhang, Luhuan Wang, Qimei Liu, Yuying Liang, Jiayu Zhang, Yunyun Xue, Yuexia Tian, Huiqi Zhang, Na Li, Cong Sheng, Pingping Nie, Suping Feng, Boshou Liao, Dongmei Bai

**Affiliations:** ^1^ Institute of Industrial Crops, Shanxi Agricultural University, Taiyuan, China; ^2^ State Key Laboratory of Sustainable Dryland Agriculture, Shanxi Agricultural University, Taiyuan, China; ^3^ College of Agronomy, Shanxi Agricultural University, Taigu, China; ^4^ College of Plant Protection, Shanxi Agricultural University, Taigu, China; ^5^ Institute of Plant Protection, Jiangsu Academy of Agricultural Sciences, Nanjing, China; ^6^ College of Life Sciences, Zaozhuang University, Zaozhuang, China; ^7^ College of Food Science and Engineering, Hainan Tropical Ocean College, Hainan, China; ^8^ The Key Laboratory of Biology and Genetic Improvement of Oil Crops, Ministry of Agriculture and Rural Affairs, Oil Crops Research Institute of the Chinese Academy of Agricultural Sciences, Wuhan, China

**Keywords:** peanut, whole genome re-sequencing, cold tolerance, germination, QTL, candidate genes

## Abstract

Low temperatures significantly affect the growth and yield of peanuts. Temperatures lower than 12 °C are generally detrimental for the germination of peanuts. To date, there has been no report on precise information on the quantitative trait loci (QTL) for cold tolerance during the germination in peanuts. In this study, we developed a recombinant inbred line (RIL) population comprising 807 RILs by tolerant and sensitive parents. Phenotypic frequencies of germination rate low-temperature conditions among RIL population showed normally distributed in five environments. Then, we constructed a high density SNP-based genetic linkage map through whole genome re-sequencing (WGRS) technique and identified a major quantitative trait locus (QTL), qRGRB09, on chromosome B09. The cold tolerance-related QTLs were repeatedly detected in all five environments, and the genetic distance was 6.01 cM (46.74 cM - 61.75 cM) after taking a union set. To further confirm that qRGRB09 was located on chromosome B09, we developed Kompetitive Allele Specific PCR (KASP) markers for the corresponding QTL regions. A regional QTL mapping analysis, which was conducted after taking the intersection of QTL intervals of all environments into account, confirmed that qRGRB09 was between the KASP markers, G22096 and G220967 (chrB09:155637831–155854093), and this region was 216.26 kb in size, wherein a total of 15 annotated genes were detected. This study illustrates the relevance of WGRS-based genetic maps for QTL mapping and KASP genotyping that facilitated QTL fine mapping of peanuts. The results of our study also provided useful information on the genetic architecture underlying cold tolerance during germination in peanuts, which in turn may be useful for those engaged in molecular studies as well as crop improvement in the cold-stressed environment.

## 1 Introduction

Peanut crops (*Arachis hypogaea* L.), which are cultivated for their usefulness as oil plants and cash crops, play an essential role as a source of edible vegetable oil as well as a leisure food. However, in high-latitude or high-altitude areas, also termed “cold areas,” coldness is an important stressor that limits the growth as well as yield of crops, including peanuts. Reportedly, global agricultural production, with the exception of typically tropical areas, may be affected by various cold-induced adverse effects, that may limit the planting range of crops, reduce crop yield and quality, and even cause crop failure. Annually estimated worldwide crop losses caused by cold stress amount to hundreds of billions of dollars (Jeon and Kim, 2013; Barrero-Gil and Salinas, 2018). Therefore, improving cold tolerance in peanuts for the betterment of peanut crops deserves to be considered as an urgent issue that requires a rapid resolution.

As thermophilic crops, peanuts require relatively high temperatures throughout their development (Wang et al., 2003). Cold injury causes different degrees of damage to peanuts during germination, seedling emergence, flowering, maturity, and other key growth stages (Zhang X, et al., 2022). Among these, damage at the germination stage is known to be a common occurrence. Shorter durations of cold stress delay the emergence of seedlings, while slightly longer durations may seriously damage the viability of germinated seeds, causing mold invasion, necrosis, and seed rotting, resulting in a lack of seedlings and ridge cutting, which seriously affects yield and quality (Bai et al., 2018). In view of the harmful effects exerted by cold stress on peanuts, with particular reference to peanut cultivation in cold regions, developing high-yielding peanut varieties with strong cold tolerance may help broaden planting areas, improve yield per unit area, and ensure product quality.

Cold tolerance of crops refers to the adaptability of crops to low-temperature environments, including tolerance to cold stress and the ability to quickly resume growth, once cold stress is removed. At present, studies investigating cold tolerance in germinating peanuts are mainly focused on the identification and screening of different germplasms for cold tolerance, differential physio-biochemical changes, and the metabolic response of peanuts to cold stress (Upadhyaya et al., 2009; Bai et al., 2018; Patel et al., 2022). The research methods used by these studies are mainly centered on phenotype identification as well as physiological and biochemical analysis. However, the advances in molecular biology-related technologies have caused some studies to shift their focus onto preliminary research on peanut cold tolerance at the molecular level. Wang et al. (2013) analyzed differentially expressed genes (DEGs) in peanuts subjected to normal and low-temperature treatments and obtained two genes that are specifically expressed in relation to cold tolerance at the germination stage. Bai et al. (2018) used 90 pairs of simple sequence repeat (SSR) polymorphic primers to evaluate the cold tolerance of 72 peanut varieties at the germination stage and found a wide variety of variations. Chang et al. (2019) screened four cold-tolerant and four cold-susceptible cultivars, for indicators of seed germination and physiological indices of seedlings at 4 °C and found that the contents of soluble sugar, soluble protein, and free proline were mildly reduced in cultivars with strong cold tolerance. However, elucidation of peanut cold tolerance is easily affected by identification methods, environment, season, climate, and other factors. In general, existing research, which is centered on germplasm exploration and genetic traits associated with cold tolerance at the germination stage, appears to lack systematism, and has failed to either produce any breakthroughs, or fine map quantitative trait locus (QTLs), or identify candidate genes, thereby severely limiting the progress of cold tolerance breeding in peanuts.

Effective molecular marker-assisted breeding for cold tolerance in peanuts involves identifying QTLs closely linked to target traits. At present, some progress has been made in QTL mapping of cold tolerance during the germination stage, in crops such as rice (Shakiba et al., 2017; Yang et al., 2020), maize (Hu et al., 2016; Li et al., 2018), and sorghum (Knoll et al., 2008; Upadhyaya et al., 2016). Continuous and rapid improvements in high-throughput sequencing technology have resulted in peanut genomic research making rapid progress. Genome sequence analysis of two wild diploid peanuts (*A. duranensis* and *A. ipaensis*) was completed in 2016 (Bertioli et al., 2016; Chen et al., 2016). In 2018, the genome sequencing analysis of *A. monticola*, an allotetraploid wild peanut, was completed (Yin et al., 2018); In 2019, a major breakthrough was made in the genome sequencing of cultivated heterotetraploid peanuts. Genomic data of the Chinese peanut varieties, *A. hypogaea* cv. Shitouqi (Zhuang et al., 2019) and *A. hypogaea* cv. Fuhuasheng (Chen et al., 2019), and the American peanut variety, *A. hypogaea* cv. Tifrinner (Bertioli et al., 2019) have been published, and these are widely used in relevant research. These advances have greatly enriched genomic information pertaining to peanuts, and moreover, various high-throughput genotyping techniques (Semagn et al., 2014; Clevenger et al., 2017; Guo et al., 2019) have made continuous progress by effectively promoting the discovery and localization of QTLs of several peanut traits. Liu et al. (2020) identified three main QTLs that regulate oil content. Khedikar et al. (2017) obtained the main QTL related to the fruit-setting number per plant, productivity, 100 fruit weight and other traits. Luo et al. (2018) successively detected major QTLs with stable pod size and kernel yield using a recombinant inbred line (RIL) population, and subsequently developed efficient Kompetitive Allele Specific PCR (KASP) markers. Huang et al. (2016) used SSR and transposon markers to map the main QTL related to aflatoxin resistance. Luo et al. (2019) identified the main QTL associated with bacterial wilt resistance in multiple environments. Han S, et al. (2018) successfully identified QTLs regulating resistance to early and late leaf spot diseases, while Agarwal et al. (2019) identified a major QTL associated with resistance to the tomato spotted wilt virus in peanuts. Thus, current progress in the development of molecular markers for important peanut traits, mentioned above, has provided a theoretical basis as well as a technical guarantee for those exploring QTLs associated with cold tolerance in peanuts. Further, RIL populations may be a better option for generating replicated and multi-environmental phenotyping data in variable space and time, that may help decipher environmental effects on target traits. Such phenotyping data may enable the detection of stable and consistent QTLs, as well as the subsequent detection of linked markers that may be useful for breeding purposes. Based on the above considerations, this study describes the development of a RIL population that can be utilized for genotyping and multi-environment phenotyping data linked to cold tolerance during germination in peanuts. The results of our study may expectedly help develop a better understanding of trait locus mapping, leading to the successful identification of genomic regions regulating cold tolerance.

## 2 Materials and methods

### 2.1 Plant materials and development of RIL population for cold tolerance

**Figure 1:**
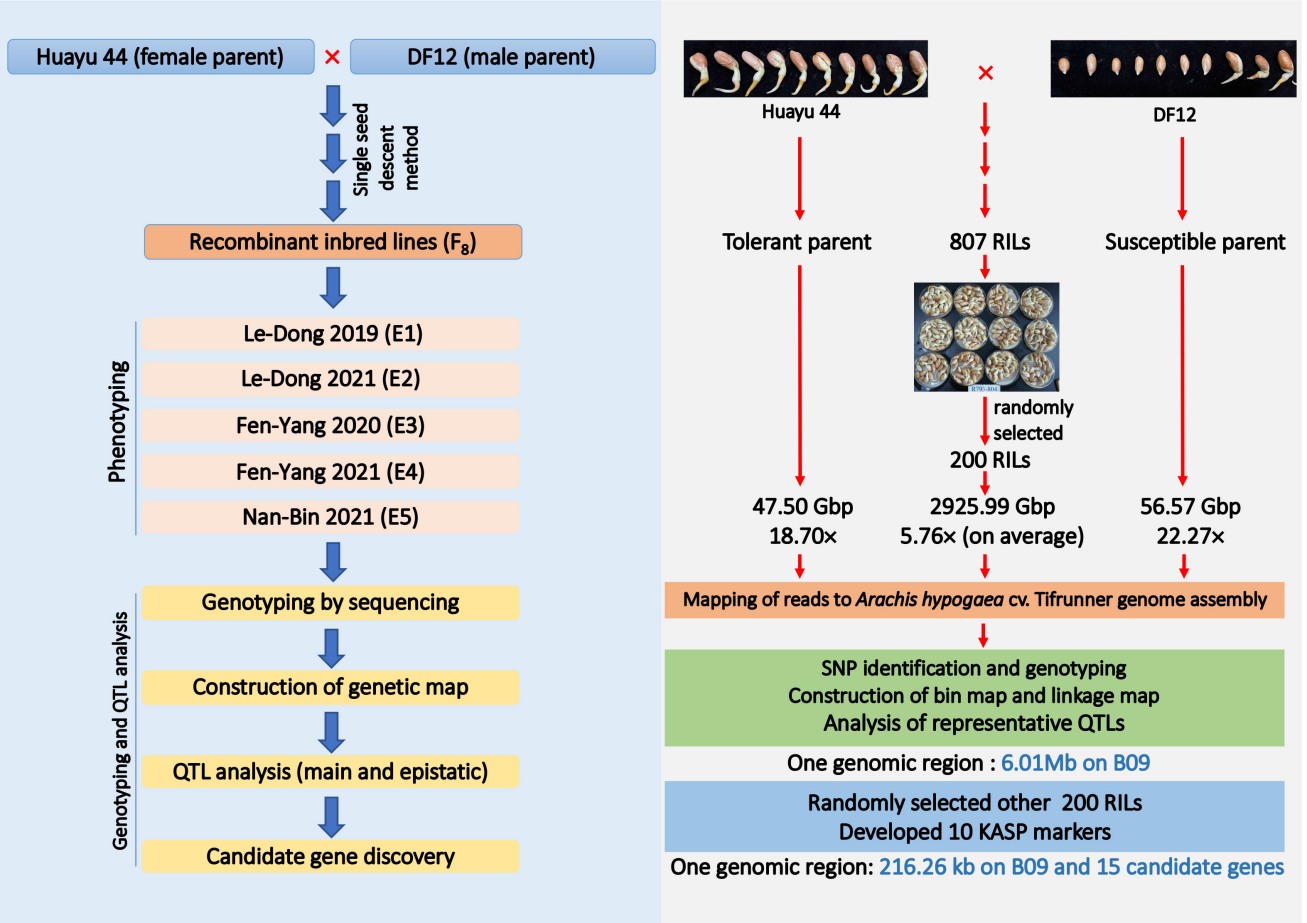
Flow chart for population development, high-density genotyping and multi-environments phenotyping.

The RIL population developed from DF12 (cold-susceptible, male parents) × Huayu 44 (cold-resistant, female parents) at the Institute of Industrial Crops, Shanxi Agricultural University, Taiyuan, China, comprised 807 individuals RILs. Huayu 44, an inter-group hybrid cultivar of the peanut genus, was bred by Shandong Peanut Research Institute (CAAS) using the incompatible wild species *Arachis glablata* Benth. DF12 is a breeding line with high oleic acid content and derived from Kainongxuan 01-6 and Baisha 1016 cross and was bred by the Industrial Crops Research Institute of the Henan Academy of Agricultural Sciences. The single seed descent (SSD) method was used until the F_8_ generation to develop the RIL population. The entire RIL population and their parents were planted in five different environments. Those grown at the Le-Dong (LD) experimental station (N18 43′ and E109 00′) in 2019 and 2021 were referred to as E1 and E2, respectively. In 2020, those used for the experiment conducted at the Fen-Yang (FY) experimental station (N37 42′ and E111 79′), were referred to as E3. In 2021, the experiments were conducted at the FY and Nan-Bin (NB) experimental stations (N18 38′ and E109 21′), and those grown there were referred to as E4 and E5, respectively (**Figure 1**).

### 2.2 Phenotyping for cold tolerance-related traits

Based on previous identification techniques and standards for peanut cold tolerance at the germination stage established by us (Bai et al., 2022), surface-sterilized seeds were incubated in water-soaked filter paper in Petri dishes, each of which contained 20 seeds. Germination experiments were performed in a growth chamber at a relative humidity of 70% and a temperature of 12°C for 3 d, then at 2°C for 3 d, and finally at 25°C for 3 d to recovery treatment. Another set was grown in a growth chamber at 25°C, as a control. The seeds were tested three times each time for replication purposes. The average value of three replicates was used as the statistical unit. The factors measured were defined as follows:


(1) Germination standard = radicle length equal to or greater than seed length;

(2) Germination rate (GR) = the number of normally germinated seeds on day 4 at 25°C/number of tested seeds × 100%;

(3) Relative germination rate (RGR) = germination rate after low temperature treatment/control germination rate × 100%.

SPSS 25.0 was used to analyze each variation coefficient and descriptive statistics.

### 2.3 DNA extraction and whole genome re-sequencing

Seed buds of the RIL population were collected and used to extract high-quality genomic DNA using the CTAB method with some modifications (Tamari et al., 2013). DNA quality and quantity were checked on 0.8% agarose using a Qubit 2.0 fluorometer (Thermo Fisher, USA). Whole genome re-sequencing (WGRS) was performed for two hundred RILs randomly selected from the 807 individuals for simultaneous single nucleotide polymorphisms (SNPs) discovery and genotyping of the mapping population. For this purpose, the DNA of each sample was fragmented into approximately 350 bp DNA fragments using a Covaris S2/E210 focused ultrasonicator (Covaris, Woburn, MA, United States). Sheared DNA was end-repaired and a single nucleotide (A) overhang was subsequently added to the repaired fragments using Klenow Fragment (3´→ 5´ exo–) (NEB, United States) and dATP at 37°C, following which barcodes and Illumina sequencing adapters were ligated to the A-tailed fragments using T4 DNA ligase (Thermo Fisher Scientific, Inc., USA). Sequence depth of the two parents was approximately 20X, while that of 200 RIL was approximately 5X on average. Polymerase chain reaction (PCR) was performed using diluted shearing-ligation DNA samples, dNTP, Q5^®^ High-Fidelity DNA Polymerase, and PCR primers. The PCR products were then purified using Agencourt AMPure XP beads (Beckman Coulter, High Wycombe, UK) and pooled. Next, the pooled samples were separated using 2% agarose gel electrophoresis. Fragments of 350 bp (with barcodes and adaptors) were excised and purified using a QIAquick gel extraction kit (Qiagen, Hilden, Germany). The gel-purified products were then diluted for pair-end sequencing (each end 150 bp) on an Illumina Novaseq 6000 system using a standard protocol (Illumina, Inc., San Diego, CA, USA).

### 2.4 SNP identification and genotyping

Low-quality reads (quality score< 20e) were filtered out, and raw reads were sorted into each progeny according to barcode sequences. After the barcodes were trimmed from each high-quality read, clean reads from the same sample were mapped to the cultivated peanut genome sequence (*A*. *hypogaea* cv. Tifrunner; https://peanutbase.org/) using Burrows-Wheeler Aligner software v.0.6.1 (Li et al., 2009). Samtools v.1.15.1 (Li and Durbin, 2009) was used for mark duplicates, while GATK v.4.1.9 (McKenna et al., 2010) was used for local realignment and base recalibration. An SNP set was formed by combining GATK and Samtools (Li and Durbin, 2009) SNP calling analysis with default parameters. SNPs identified between parents were homozygous, and only biallelic SNPs were regarded as polymorphic, and the marker integrity over 80% was maintained for bin calling later. Genotypes of the RIL were recognized at this SNP site.

### 2.5 Bin map and linkage map construction

Based on the SNP VCF matrix, SNPBinner v.0.1.1 software (Gonda et al., 2019) which uses the Hidden Markov Model (HMM) method to calculate recombination breakpoints, was employed to build co-separation bin markers. Based on their location in the peanut genome, marker loci were partitioned primarily into linkage groups (LGs). The linkage map was constructed from recombination bins serving as genetic markers using Icimapping v.4.1 using model 2-opt, and window size=5 for rippling (Meng et al., 2015). Map distances were estimated using the Kosambi mapping function (Kosambi, 2012). The genetic linkage map, based on the reference genome, was visualized using the R package in LinkageMapView v.2.1.2 (Ouellette et al., 2018). All sequences of bin markers that were constructed in the linkage map were aligned back to the physical genome sequence of Tifrunner using BLAT software v.36x5 (Kent, 2002) to confirm their physical positions in the genome. The Spearman correlation coefficient was calculated in order to evaluate the collinearity between the genetic map and the reference genome.

### 2.6 QTL analysis and fine mapping *via* KASP-based genotyping

QTLs were identified using an interval mapping model implemented *via* the Complex interval mapping (CIM) in R/qtl package (Arends et al., 2010). The logarithm of odds (LOD) threshold was determined by applying 1000 permutation tests with 1% probability for each trait. Subsequently, KASP genotyping was performed, and 10 SNP markers were developed inside the main QTL as well as on its sides. Two hundred RILs from 807 lines that did not participate in WGRS were randomly selected and genotyped. Based on genotyping results and their corresponding RGR phenotypes, a regional genetic map and QTL analysis were performed using IciMapping v.4.1 (https://www.isbreeding.net/), according to Liu’s method (Liu et al., 2019).

## 3 Results

### 3.1 Phenotypic variation of cold tolerance-related traits in the RILs and their parents

**Figure 2:**
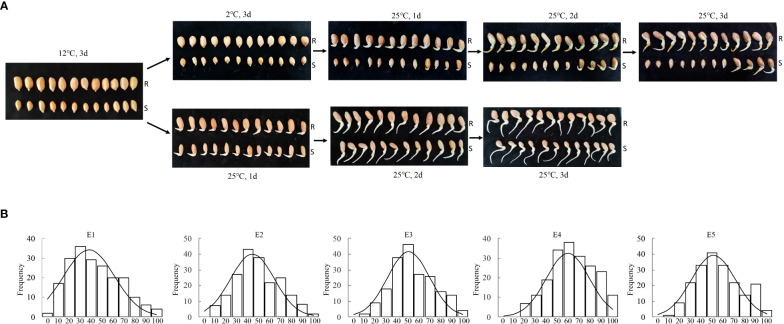
Phenotypic variation of cold tolerance-related Traits in RILs and their parents. **(A)** Germination experiments were performed in a growth chamber at a relative humidity of 70% and a temperature of 12 °C for 3 d, then at 2 °C for 3 d, and finally at 25 °C for 3 d to recovery treatment. The parents showed distinct differences in cold tolerance. The radicle lengths of almost all tested Huayu 44’s seeds were greater than their seed lengths. By contrast, the radicle lengths of most DF12’s seeds were either equal to or less than their seed lengths, while some seeds failed to germinate. R, female parent Huayu 44; S, male parent DF12. **(B)** Frequency distribution for relative germination rate in RIL population at E1, E2, E3, E4 and E5. Instrument-measured traits in all five environments followed normally distributed.

The relative germination rates of two hundred RILs and their parents from five environments were tested, respectively. The parents “Huayu 44” and “DF12” showed distinct differences in cold tolerance (**Figure 2A**; **Table S1**). The radicle lengths of almost all tested Huayu 44’s seeds were greater than their seed lengths. By contrast, the radicle lengths of most DF12’s seeds were either equal to or less than their seed lengths, while some seeds failed to germinate. The average relative germination rates of Huayu 44 and DF12 were 97.42%, and 38.63%, respectively. The range of variation in the relative germination rates of the RIL populations was large (0–96.55%) (**Table S1**). The types of population variation were relatively rich, with a coefficient of variation of more than 33%. Instrument-measured traits in all five environments followed normally distributed (**Figure 2B**; **Table S2**).

### 3.2 Variation calling and annotation

**Figure 3:**
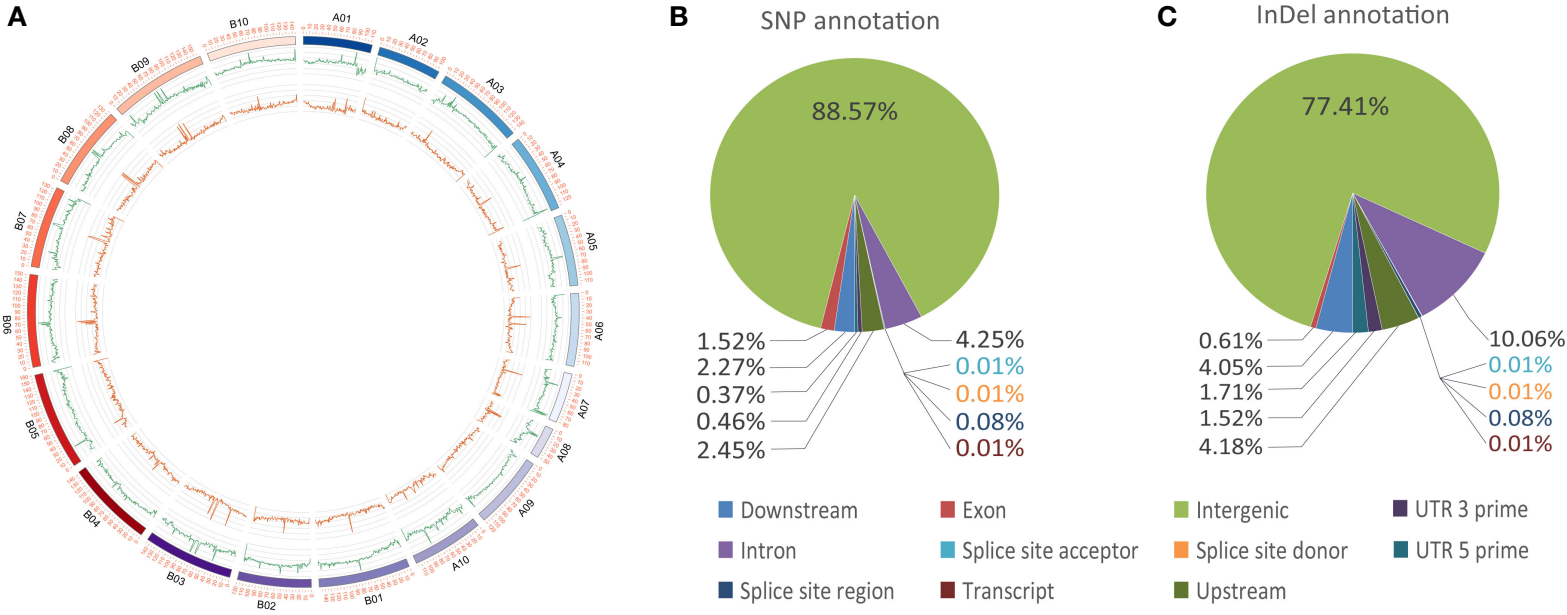
Genome variations and annotations. **(A)** Circos plot of SNP and InDel distributions. The outer ring indicates the SNP distribution, whereas the inner ring indicates the InDel distribution. **(B)** Pie charts of SNP annotations. **(C)** Pie charts of InDel annotation information.

Whole genome re-sequencing of the female parent, male parent and all the two hundred individuals generated a total 47.50, 56.57, and 2925.99 Gb of clean data with Q30 ≥ 84%, representing, on average, sequencing depths of 18.70×, 22.27×, and 5.76×, respectively (**Figure 1**; **Table S3**). All clean reads were mapped to the genome of the cultivated peanut Tifrunner, where the mapping rates were 99.07% for Huayu 44, 98.80% for DF12 and 99.35% for the offspring on average. A total of 447,528 SNPs and 220,443 InDels were called between the parents (**Figure 3A**). Most SNPs (88.57%) were annotated in the intergenic regions, whereas 1.52% were annotated in the exon regions (**Figure 3B**). With respect to InDels, 77.41% were annotated in intergenic regions while 0.61% were located in exon regions (**Figure 3C**). This map lays a foundation for further bin establishment and candidate genes speculation.

### 3.3 Bin map and genetic map construction

**Figure 4:**
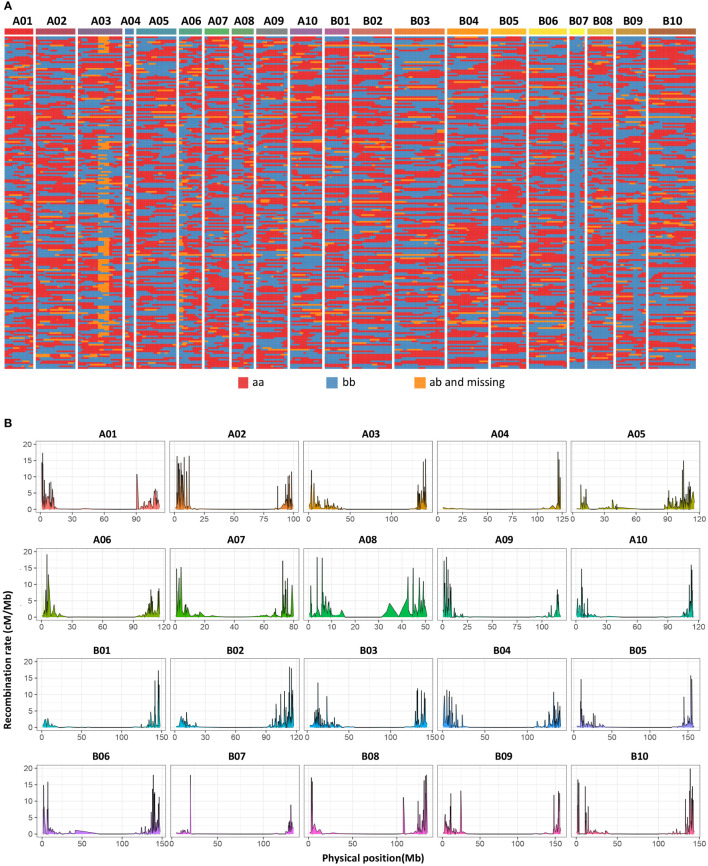
Bin map and heatmap of the RIL population. **(A)** A total of 2494 bins were inferred from resequencing-based high-quality SNPs. Red indicates DF12’s background, and blue indicates Huayu 44’s background. Yellow indicates heterozygous genotypes and missed genotypes. **(B)** Genome-wide recombination hotspots in the 20 peanut chromosomes.

**Figure 5:**
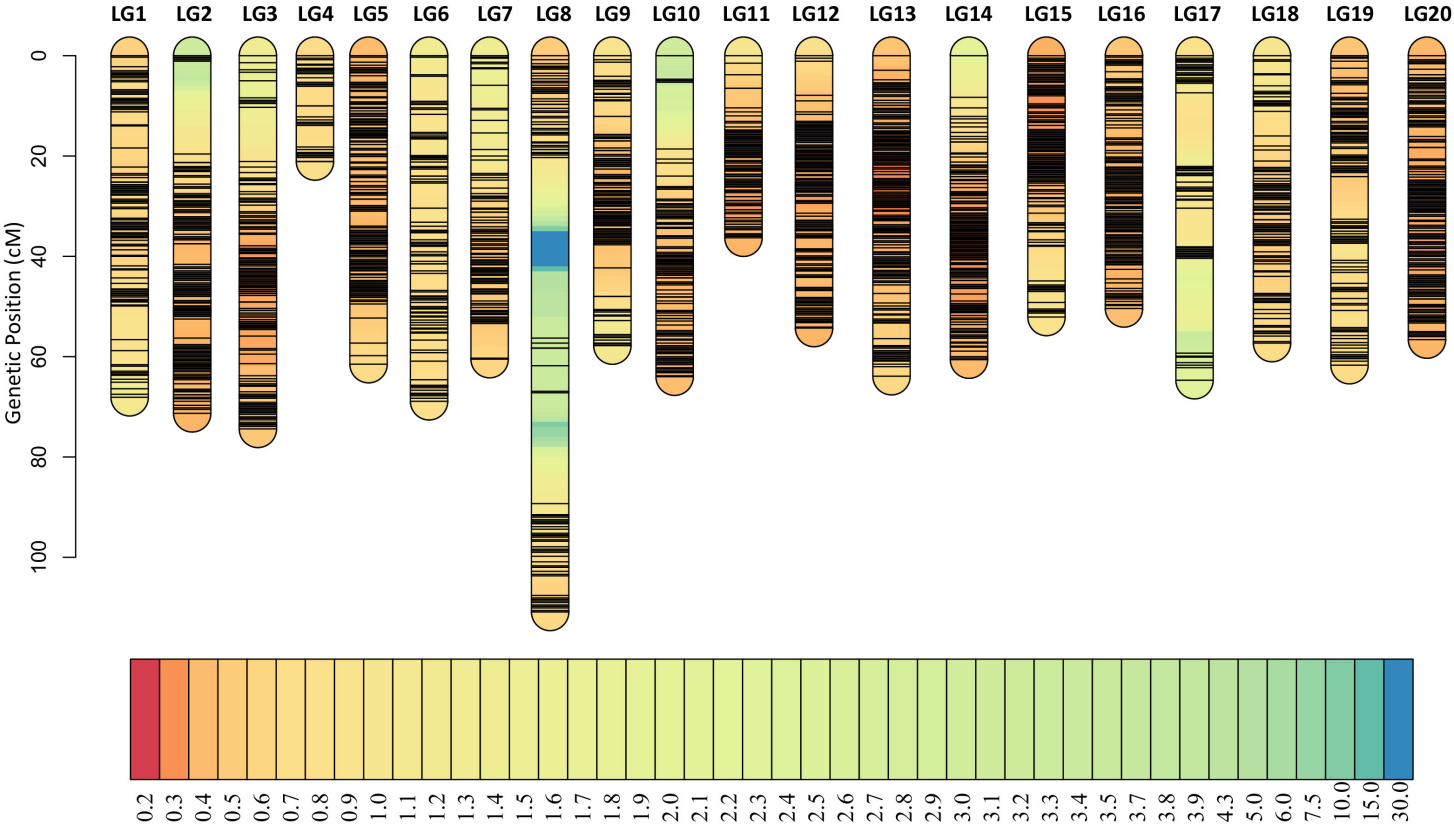
High-density genetic map. A high-resolution and high-quality genetic map had been constructed for QTL mapping. The short black lines on linkage groups mean the locus of bin markers.

Among all the SNPs, 166,274 are parental polymorphic and homozygous (aa×bb), and these SNPs are used for bin calling. Based on the NMM method, a bin map representing recombination intervals was generated, and 2494 bins were obtained on 20 chromosomes (**Figure 4A**; **Table S4**). The mean bin number was approximately 125, with the highest number being approximately 194 on chromosome B03 and the lowest number being approximately 34 on chromosome A04. The basic information statistics of the number of bins, distance, MaxGap, and Spearman of each LG are shown (**Tables S5** and **S6**). The largest and minimum bin MaxGap belonged to chromosomes B07 (18.9 cM) and B06 (1.99 cM), respectively. On average, there were 67 SNPs within a bin. This set of bin markers was used to construct a high-density genetic map including 20 linkage groups (**Figure 5**). LG8 had the largest genetic distance (110.90 cM), while the smallest genetic distance (21.08 cM) was observed for LG4. For LG8, the largest gap stretched across 36 cM. The ratio of the genetic distances between adjacent markers< 5 cM across all LGs approached 93% (**Table S5**). Moreover, collinearity was high between the genetic map and the reference genome (**Table S6**; **Figure S1**). The recombination hotspot (RH) analysis revealed most RHs were unequally distributed across all 20 LGs, most of which were located on the arm of corresponding chromosomes (**Figure 4B**). The sources of the larger segments in each individual were consistent, indicating that the quality of the genetic map was very high. The evaluation of the linkage relationship showed that the linkage relationship between adjacent markers on each LG was very strong. With the increase of distance, the linkage relationship between markers as well as that between distant markers gradually weakened, indicating that the sequence of markers was correct (**Figure S1**). These genomic and genetic indicators indicated that a high-resolution and high-quality genetic map had been constructed for QTL identification.

### 3.4 QTL identification for cold tolerance-related traits

**Figure 6:**
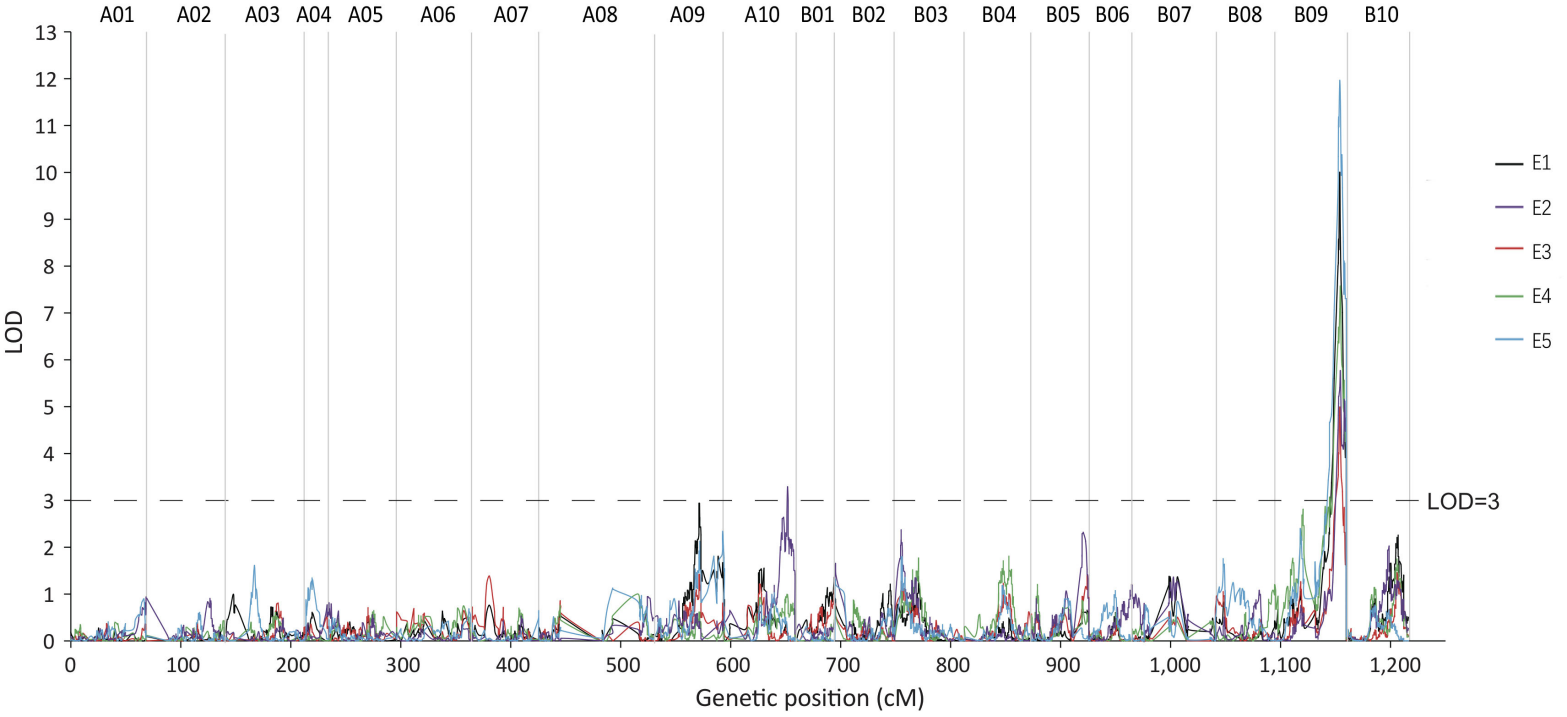
The germination stage cold-related QTL *qRGRB09* is located on chromosome B09. *qRGRB09* on chromosome B09 was stably detected in all five environments.

QTL mapping was performed using CIM in R/qtl software, while ggplot^2^ was used to map LOD linkage distribution. The LOD significance threshold (p< 0.01) was calculated using a Permutation Test (1000 times), and *qRGRB09* on chromosome B09 was stably detected in all five environments after taking a union set, with a LOD ranging from 4.99 to 11.96, a phenotypic variation explained (PVE) ranging from 10.85 to 24.07%, and an additive effect value ranging from 6.26 to 10.27. The maximum phenotypic variation contribution rate and additive effect value were 24.07% and 10.27, respectively. According to the regional linkage analysis, the five QTLs were located between 49.70–61.75 cM (4.23 Mb), 54.23–61.75 cM (3.81 Mb), 54.77–56.37 cM (755.85 kb), 50.78–61.75 cM (4.07 Mb), and 46.74–61.75 cM (6.01 Mb) in E1, E2, E3, E4, and E5, respectively (**Figure 6**; **Table 1**). The physical distance of *qRGRB09* was 6.01 Mb (46.74–61.75 cM) after taking a union set. This indicated that, *qRGRB09* was a major QTL site that was not sensitive to the environment and regulated cold tolerance during germination in peanuts. These findings suggested that *qRGRB09* may be useful for determining the KASP mark for interval design.

**Table 1:** QTLs detected in the RILs population using the high-resolution genetic map.

Environment	QTL	Chromosome	LOD Threshold	Max LOD	Low Marker	High Marker	Low Position (cM)	High Position (cM)	Physical Interval	Additive effect	PVE (%)
E1 (2019LD)	*qRGRB09*	B09	3.97	10.00	C19P153976706	C19P157324174	49.70	61.75	4.23 Mb	10.27	20.57
E2 (2021LD)	*qRGRB09*	B09	4.06	5.76	C19P154454417	C19P157324174	54.23	61.75	3.81 Mb	7.03	12.43
E3 (2020FY)	*qRGRB09*	B09	3.93	4.99	C19P154855110	C19P155555990	54.77	56.37	755.85 Kb	6.26	10.85
E4 (2021FY)	*qRGRB09*	B09	4.24	7.57	C19P154187296	C19P157324174	50.78	61.75	4.07 Mb	7.77	15.99
E5 (2021NB)	*qRGRB09*	B09	3.92	11.96	C19P152409354	C19P157324174	46.74	61.75	6.01 Mb	9.77	24.07
Average	*qRGRB09*	B09	4.01	13.44	C19P153065061	C19P157324174	47.01	61.75	5.43 Mb	8.07	26.61

PVE, phenotypic variation explained; LOD, logarithm of odds.

### 3.5 Fine mapping and prediction of candidate genes on chromosome B09

**Figure 7:**
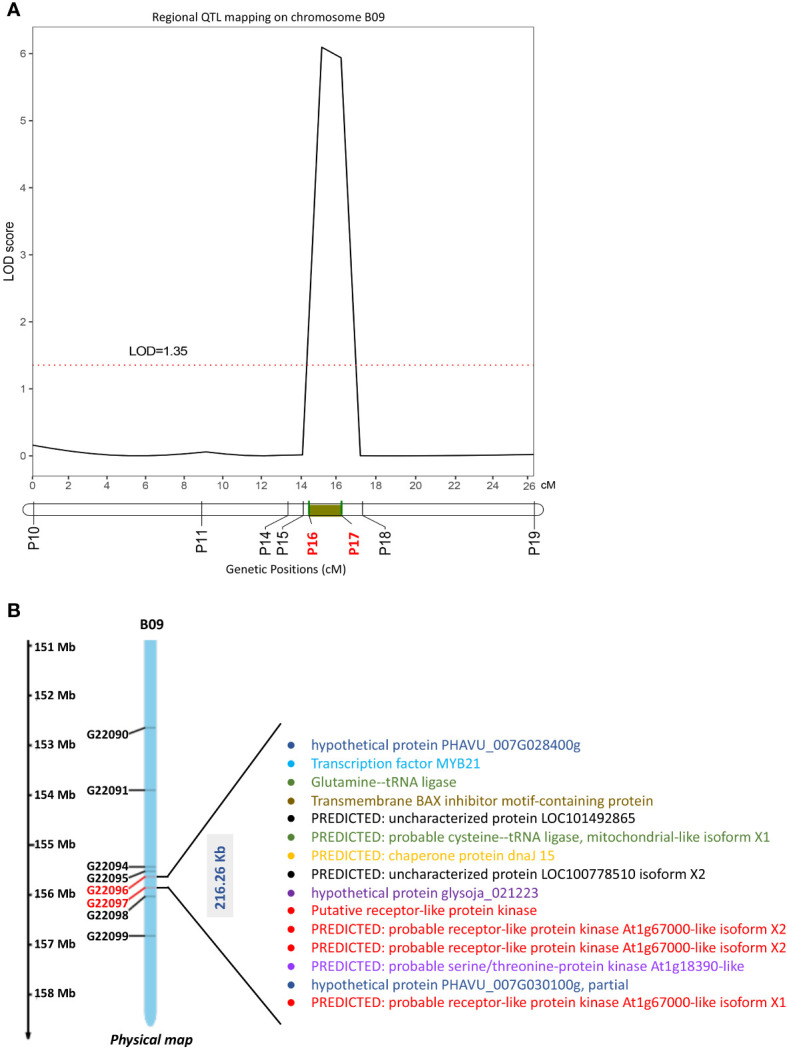
Fine mapping of cold-related QTLs and candidate genes screen. **(A)** KASP-based regional linkage analysis to narrow the region QTL (*qRGRB09*) using IciMapping V4.1 in the RIL population (black curve). P16, KASP marker G22096; P17, KASP marker G22097. **(B)** Candidate genes identified in 216.26 Kb QTL region mapped on chromosome B09. This region was rich in RLKs, tRNA ligase, MYB, BI1-like protein, *DnaJ*, serine/threonine-protein kinase, and other uncharacterized proteins.

We found 64 SNP sites suitable for KASP and then developed 10 evenly distributed KASP markers (**Table S7**) covering the QTL regions (chrB09:152644698–156822195) detected in the RIL population, to further confirm and narrow the *qRGRB09* on chromosome B09. Eight of the 10 markers were successfully genotyped (**Figure S2**). When intersection of QTL intervals of all environments was taken into account, the regional QTL mapping analysis placed *qRGRB09* between KASP markers G22096 (P16) and G22097 (P17) (chrB09:155637831–155854093) spaced 14.57–16.35 cM apart, with a 6.10 LOD and 11.55% PVE and a physical length of 216.26 kb (**Figure 7A**; **Table S8**). Annotation information was obtained to mine causal genes in the above mentioned 216.26 kb region. Fifteen annotated genes, including *Arahy.GJ31ZB*, *Arahy.RK8HN4*, *Arahy.VKD07K*, *Arahy.YV67XB*, *Arahy.9KP105*, *Arahy.A245TJ*, *Arahy.28TJ89*, *Arahy.NA36PT*, *Arahy.MQZQ11*, *Arahy.12LLDS*, *Arahy.Z64X3Y*, *Arahy.GW3QL7*, *Arahy.HNK57V*, *Arahy.H41NY2*, and *Arahy.2U2D57*, were observed (**Figure 7B**; **Table S9**). These candidate genes encoded receptor-like protein kinases (RLKs), tRNA ligase, MYB transcription factor, BAX inhibitor motif-containing protein (BI1) -like protein, Chaperone DnaJ-domain superfamily protein (DnaJ), serine/threonine-protein kinase, and some uncharacterized proteins. Importantly, homologs of some candidate genes have been shown to be involved in cold tolerance (Shakiba et al., 2017; Davik et al., 2021; Han et al., 2022; Li et al., 2022). Then, we examined the expression levels of *RLK*, *MYB* and *DnaJ* in two parents, respectively. The three candidate genes demonstrated uniformly increased expression level in Huayu44, while hardly any noticeable change was observed in DF12 (**Figure S3**). Further exploitation and functional investigation of these genes may contribute to a better understanding of the cold-tolerance domestication process in peanuts.

## 4 Discussion

Cold tolerance is vital for the sustainability of peanut production in cold areas. However, dedicated trait mapping or marker discovery studies aimed at elucidating the genetic basis underlying cold tolerance during peanut germination appear to be lacking. The current study, which was conducted in the above context, generated data pertaining to multi-environment phenotyping, high-quality genetic maps, QTLs, and linked markers, all of which were linked to cold tolerance during germination. The information generated by this study may provide the groundwork for the establishment of projects aimed at facilitating fine mapping, gene discovery, and marker development associated with cold tolerance in peanuts.

### 4.1 Ideal simulation condition was constructed for cold stress at the germination stage

To simulate the field conditions, the alternating low temperature regime (12°C, 3 d; 2°C, 3 d; and 25°C, 3 d recovery) was adopted during seed germination in our study. The parents showed distinct differences in cold tolerance across all five environments. In the RIL populations, the variation range of relative germination rate was large, 0.00–94.83% in E1(2019LD), 3.77–92.45% in E2 (2021LD), 7.27–94.83% in E3 (2020FY), 12.07–96.55% in E4 (2021FY), and 8.93–94.83% in E5 (2021NB) (**Table S1**), all of which were normally distributed. Types of population variation were relatively rich, with a coefficient of variation of more than 33%. The current experiments confirmed the feasibility of simulating conditions as well as constructing successful RIL populations. Therefore, the above-stated simulation conditions at the germination stage should provide the ideal stage for phenotyping for the purpose of genetic mapping and for making reliable selections in breeding programs aimed at developing tolerant varieties.

### 4.2 Re-sequencing-based bin map strategy facilitated the mapping resolution

WGRS has accelerated the process of gene mapping and cloning in crops. The high-density genetic maps based on re-sequencing constructed by bin markers have shown that continuous SNPs without recombination in one bin marker have been realized in many crops (Li et al., 2016; Lu et al., 2017; Han K, et al., 2018; Tong et al., 2020), including leguminous species (Agarwal et al., 2018; Agarwal et al., 2019; Garg et al., 2022). In General, a population containing 100–400 individuals harbors 1000–4000 bins (Zhang H, et al., 2022), indicating that the QTL mapping resolution for a species with a genome size above 2 Gb may approximate hundreds of kilobases (Zhang et al., 2022). We obtained 2494 bins in total with 1008 kb per bin on average (**Figure 4A**; **Table S4**), where four out of five QTL regions were under 5 Mb (**Table 1**), indicating that a high mapping resolution had been achieved. Moreover, we believe that sequencing‐based genotyping also enhances mapping resolution and facilitates the development of high-quality genetic maps. The present study developed a high-quality genetic map with 447,528 SNP loci. The tetraploid genome of cultivated peanuts with a narrow genetic base poses a challenge when attempting to achieve optimum genetic density. High-marker density genetic maps are essential for high-resolution genetic mapping and marker discovery.

### 4.3 A novel and consistent fundamental QTL for further fine mapping

Following the release of peanut genome assemblies, many studies have reported quantitative variations in a diverse range of traits (Agarwal et al., 2018; Luo et al., 2018; Agarwal et al., 2019; Luo et al., 2019). Cold tolerance during germination is important for many plants. At present, many studies focused on the QTL mapping of this trait have been conducted. In rice, a high-density genetic map detected six QTLs, which explained 5.13–9.42% of the total PVE during the germination stage and different genetic loci that regulated cold tolerance at germination and bud stages (Yang et al., 2020). Thapa et al. (2020) identified 31 distinct QTL regions and 13 QTL regions in the japonica subset, and 7 distinct QTL regions in the indica subset, underlying cold tolerance during germination in rice. Hu et al. (2016) performed QTL analysis using 243 IBM Syn4 RILs and detected six QTLs associated with a low-temperature germination rate. Li et al. (2018) generated three connected F_2:3_ populations to detect QTLs related to seed germination ability at low temperatures and found 43 QTLs and three mQTL regions. Interestingly, our study revealed only one average region QTL (*qRGRB09*) with a high LOD (13.44) and PVE (26.61%) for cold tolerance traits, which were continuously mapped using the phenotypic data from five environments. The different results comparing the previous studies and ours might be caused by different testing conditions, such as type and number of molecular markers, mapping populations, as well as temperature simulation conditions. Anyway, our results clearly identified the B09 genome as the source of tolerance that hosted the main-effect QTL for cold tolerance, indicating that the cold tolerance mechanism for peanuts may be different from that of other crops.

### 4.4 Identification of candidate genes for cold tolerance

Using SNP-derived KASP markers, a regional genetic map rapidly mapped *qRGRB09* to a shared region of 216.26kb (chr:155637831-155854093) on chromosome B09 (**Figure 7A**; **Table S8**). The physical length of the region was slightly smaller than that of the preliminary mapping. In preliminary mapping, the physical length of QTLs was 4.23 Mb, 3.81 Mb, 755.85 kb, 4.07 Mb, and 6.01 Mb in E1, E2, E3, E4, and E5, respectively. This shows that we had successfully performed fine mapping using KASP. Based on the results of QTL analysis and the evaluation of this region with the peanut genome assembly, followed by genome annotation, identified a total of 15 genes (**Figure 7B**; **Table S9**). This region was rich in RLKs, tRNA ligase, MYB, BI1-like protein, *DnaJ*, serine/threonine-protein kinase, and other uncharacterized proteins. The leucine-rich repeat receptor-like protein kinase (LRR-RLK) gene family, which is the largest family of the receptor-like protein kinase (RLKs) superfamily in higher plants, regulates plant growth, stress responses, and signal transduction (Cheng et al., 2021). Interestingly, we identified four isoforms of RLK proteins in the 216.26Kb region which may be key candidate genes for cold tolerance. In addition, the region also contained a serine/threonine protein kinase, the homolog of which is *LRK10L1.2*, reported to be a good candidate for regulating the effects of freezing tolerance at the QTL identified in *Fragaria vesca* L. (Davik et al., 2021). MYB transcription factors are associated with the mechanism of cold tolerance in other plants (Shakiba et al., 2017; Han et al., 2022; Li et al., 2022). Reportedly, *DnaJ* co-chaperons also play a vital role in stress response and have been found to be involved in the maintenance of PSII under chilling stress and the enhancement of drought tolerance in tomato (Kong et al., 2014; Wang et al., 2014). Bax inhibitor is a conserved protein that suppresses the pro-apoptotic protein Bax and eventually inhibits cell death in plants (Huckelhoven, 2004). Some studies have shown that Bax inhibitor, a cytoprotective protein, is involved in the response to heat stress by plants and fungi (Chen et al., 2015; Lu et al., 2018). Heat-stress-induced upregulation of the Bax inhibitor leads to the upregulation of heat-response genes, such as *sHSP*, *HSP70B*, and *HSP90.1*, which enhance thermotolerance in wheat (Lu et al., 2018). These studies indicate that the BI1-like protein deserves to be considered as an important temperature-response protein, which may contribute to cold tolerance. that is, comparable expression in wild type and dcl1, but measurably reduced level in dcl4 mutant (**Figure 4A**). Our qRT results also confirm that these candidate gene, such as *RLK*, *MYB* and *DnaJ*, may be key candidate genes for future genomics-assisted improvement for cold tolerance.

## 5 Conclusion

In summary, this study successfully performed WGRS-based genotyping, leading to the creation of a high-quality genetic map for cold tolerance during peanut germination for the first time. This data was used in multi-environment phenotyping, which helped dissect the polymorphic nature of this important stress factor and facilitated the identification of one pivotal genomic region corresponding to cold tolerance in peanuts. Most importantly, a 216.26 kb QTL region on chromosome B09 comprising 15 genes, including potential cold-tolerant genes, such as RLKs, MYB and *DnaJ*, among others, was also discovered. However, further exploration aimed at dissecting the 216.26 kb region *via* constructing secondary mapping populations and assessing the potential of identified candidate genes and genetic markers that can be used in breeding *via* next-generation sequencing technologies, are felt to be warranted.

## Data availability statement

The data presented in the study are deposited in the NCBI Sequence Read Archive (SRA) repository, accession number PRJNA931845.

## Author contributions

DB and BL designed experiments. XZ, XJZ, LW, QL, YL, JZ, YX,YT, HZ, NL, CS, PN and SF performed experiments. XZ and DB performed the data analysis. XZ wrote the manuscript. All authors contributed to the article and approved the submitted version.
