# Supplementary material for: Fine mapping of a QTL and identification of candidate genes associated with cold tolerance during germination in peanut (Arachis hypogaea L.) on chromosome B09 using whole genome re-sequencing
Source: Front Plant Sci. 2023 May 8;14:1153293. doi: 10.3389/fpls.2023.1153293 (PMC10200878; doi:10.3389/fpls.2023.1153293)
Supplement: Supplementary file 1 [file DataSheet_1.pdf]

Supplementary Material

Fine Mapping of a QTL and Identification of Candidate Genes Associated with Cold Tolerance during Germination in Peanut (*Arachis hypogaea* L.) on Chromosome B09 using Whole Genome Re-sequencing

Xin Zhang[1, 2], Xiaoji Zhang[3], Luhuan Wang[3], Qimei Liu[4], Yuying Liang[3], Jiayu Zhang[3], Yunyun Xue[1], Yuexia Tian[1], Huiqi Zhang[1], Na Li[1], Cong Sheng[5], Pingping Nie[6], Suping Fing[7], Boshou Liao[8*] and Dongmei Bai[1*]

*** Correspondence:**
Dongmei Bai: baidm1221@163.com; Boshou Liao: lboshou@hotmail.com

1 Supplementary Figures and Tables

1.1 Supplementary Figures

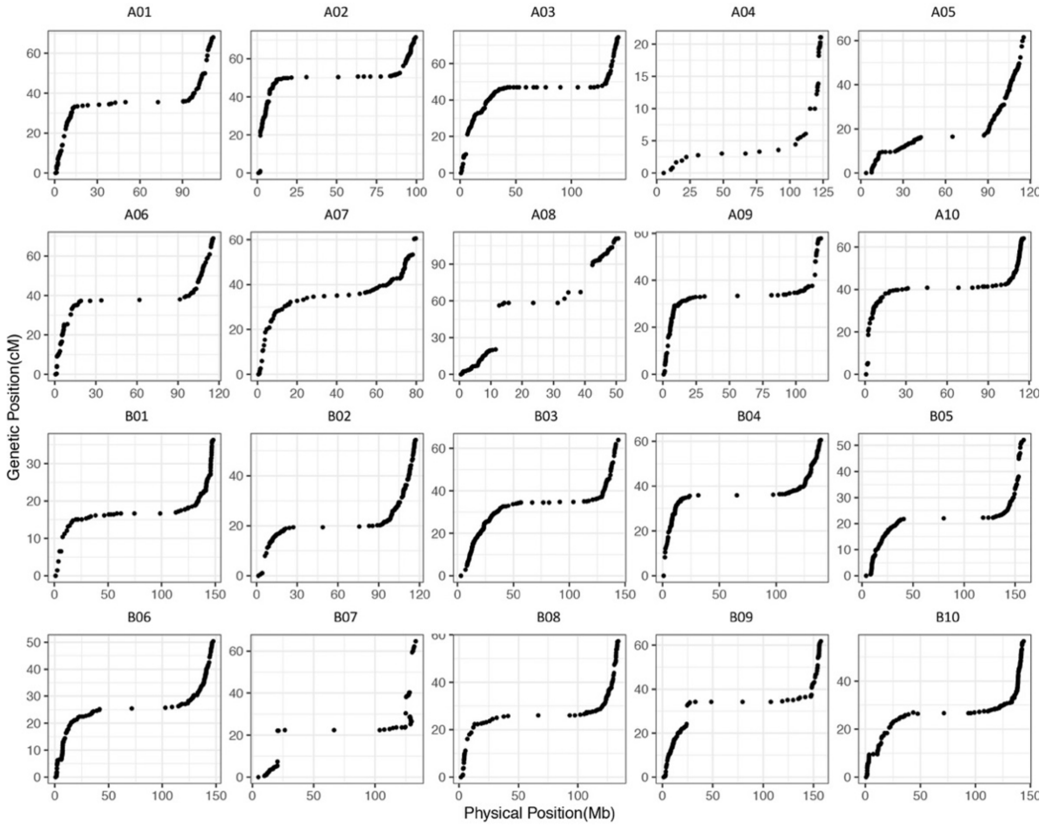

Supplementary Figure S1 Collinearity evaluation between the high-density genetic map and the reference genome assembly. high collinearity existed between the genetic map and reference genome.

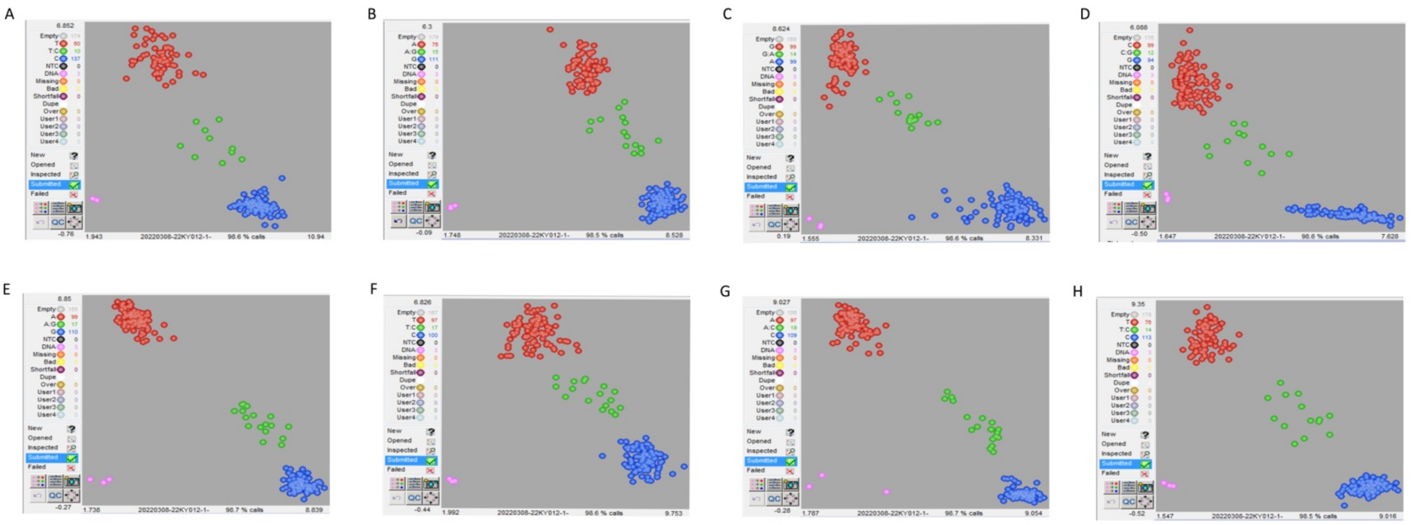

Supplementary Figure S2 Eight of the 10 markers were successfully genotyped. A, G22090. B, G22091. C, G22094. D, G22095. E, G22096. F, G22097. G, G22098. G, G22099.

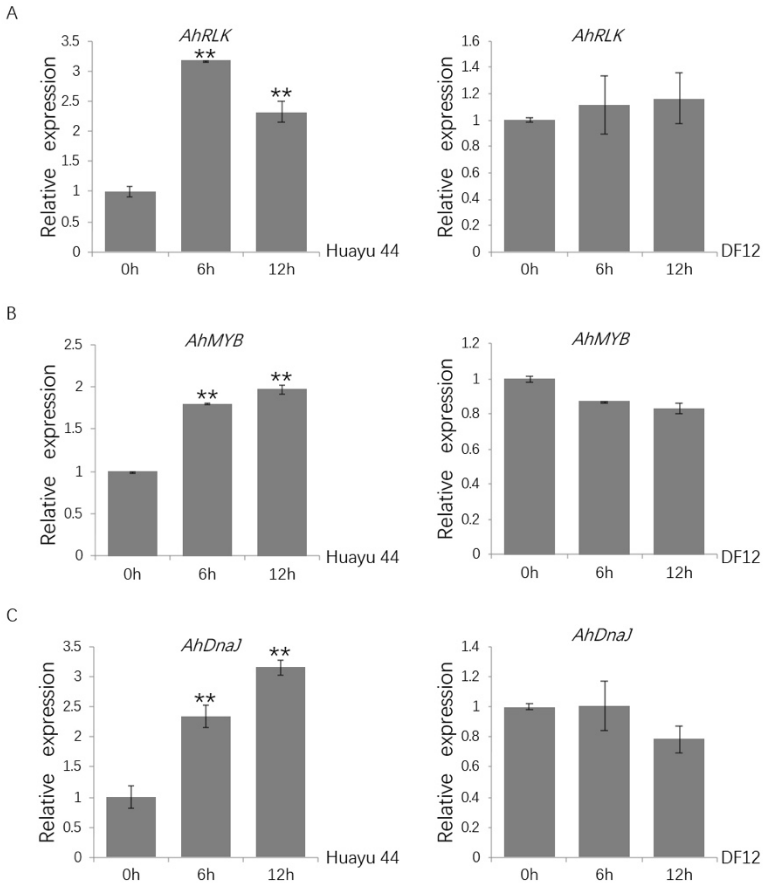

Supplementary Figure S3 Comparison of the relative expression levels of candidate genes in two parents. (A) qRT-PCR analyses of candidate gene of *AhRLK* expression levels in two parents upon low-temperature (2°C) treatment at the indicated time points, respectively. (B) qRT-PCR analyses of candidate gene of *AhMYB* expression levels in two parents upon low-temperature (2°C) treatment at

the indicated time points, respectively. (C) qRT-PCR analyses of candidate gene of *AhDnaJ* expression levels in two parents upon low-temperature (2°C) treatment at the indicated time points, respectively. Data are shown as means ± SD (n = 3). Student's *t*-test was used to determine the significance of differences between 0 h and the indicated time points. Asterisks indicate significant differences (**p<0.01). Similar results were obtained from three biological replicates.

Supplementary Tables

Supplementary Table S1 The relative germination rates of the two parents and two hundred RILs.
Supplementary Table S2 Variance analysis of cold tolerance-related traits among the two parents and RIL plants.
Supplementary Table S3 Sequencing information of the RIL population.
Supplementary Table S4 Genome wide bin marker information statistics.
Supplementary Table S5 Summary of the high density linkage groups.
Supplementary Table S6 Spearman correlation coefficient between the high density genetic map and the reference genome assembly.
Supplementary Table S7 The primers of 10 SNP-based KASP markers.
Supplementary Table S8 Fine mapping by kompetitive allelespecific PCR genotyping.
Supplementary Table S9 Candidate genes for cold tolerance-related traits.
